# First detection of *Onchocerca lupi* infection in dogs in southern Spain

**DOI:** 10.1186/s13071-016-1587-1

**Published:** 2016-05-18

**Authors:** Guadalupe Miró, Ana Montoya, Rocío Checa, Rosa Gálvez, Juan José Mínguez, Valentina Marino, Domenico Otranto

**Affiliations:** Department of Animal Health, Faculty of Veterinary Sciences, Universidad Complutense de Madrid, Madrid, Spain; Hospital Veterinario Guadiamar, Sevilla, Spain; Department of Veterinary Medicine, University of Bari, Bari, Italy

**Keywords:** *Onchocerca lupi*, Dog, Spain, Vector borne disease, Zoonosis

## Abstract

**Background:**

*Onchocerca lupi* causes ocular pathology of varying severity in dogs from south-western United States, western Europe and northern Asia. This filarioid has also been recognized as a zoonotic agent in Tunisia, Turkey, Iran and the USA, though the information about the biology and epidemiology of this infection is largely unknown. In Europe, *O. lupi* has been reported in dogs from Germany, Greece, Hungary, Portugal and Romania and in a cat from Portugal. The present study was designed to establish the occurrence of *O. lupi* in dogs in southwestern Spain.

In the present study a total of 104 dogs of different breed, sex, and age living in a shelter in Huelva (SW Spain) were examined. Skin snip samples were collected using a disposable scalpel in the forehead and inter-scapular regions and stored as aliquots in saline solution (0.5 ml) before light microscopy observation of individual sediments (20 μl) and molecular examination.

**Results:**

Of the 104 dogs examined, 5 (4.8 %) were skin snip-positive for *O. lupi:* two by microscopy and three by PCR. One of the *O. lupi* infected dogs showed neurological signs but ocular ultrasonography and/or MRI detected no abnormalities.

**Conclusions:**

This first report of *O. lupi* infection in dogs in southern Spain expands the range of geographical distribution of this parasite and sounds an alarm bell for practitioners and physicians working in that area.

## Background

Canine ocular onchocercosis caused by *Onchocerca lupi* is a zoonotic parasite, which causes ocular conditions primarily in dogs. Since its original retrieval in the sclera of a wolf (*Canis lupus*) in Georgia [1], this nematode has been detected in dogs in Hungary [2], Greece [3–6], Germany [7], Portugal [4, 8] and Romania [9]. In addition, it has been identified in both dogs and cats in the USA [10, 11], and, more recently, in a cat in southern Portugal [12].

Adult nematodes are generally embedded in granulomatous nodules in ocular retrobulbar space, orbital fascia, eyelid, third palpebra, conjunctiva and sclera [13]. Infection by *O. lupi* causes lesions ranging from no apparent clinical sings to conjunctivitis, photophobia, lacrimation, ocular discharge, keratitis, uveitis, exophthalmos and even blindness [14, 15].

Accordingly, the diagnosis of *O. lupi* relies on the detection of microfilariae in skin snips [15] and may also be investigated by the use of imaging tools (i.e. ultrasound scans, computed tomography and magnetic resonance imaging - MRI) [16].

The zoonotic potential of *O. lupi* has been demonstrated in Tunisia, Turkey, Iran and USA [17], with an increasing interest of the scientific community in investigating the definitive and intermediate hosts of this nematode as well as the range of distribution of the infection it causes. Therefore, this study aims at investigating the occurrence of canine onchocercosis in dogs from the southwestern region of Spain.

## Methods

### Study area and sample procedures

Dogs were recruited among those living in an animal shelter in the municipality of Valverde del Camino, Huelva in the Southwest of Spain (latitude 37°34'20"N; longitude: 6°45'12"W; altitude: 272 m above sea level).

One hundred and four adult dogs (older than one year) receiving no treatment against ecto- and/or endoparasites were sampled. For each animal, data on breed, age, gender, weight, health status (including ocular signs) and place of origin were recorded in a clinical file. All dogs were subjected to a thorough physical examination followed by an eye examination after the administration of anaesthetic eye drops (tetracaine hydrochloride and naphazoline hydrochloride, Colircusí Anestésico® 0.50 %, ALCON). Skin snip samples were obtained from each animal. Skin snip samples were collected using a disposable scalpel over an area of ≈ 0.2 × 0.2 × 0.2 cm from the forehead and interscapular region and stored in aliquots containing 0.5 ml of saline solution (NaCl 0.9 %) at room temperature until examination under a microscope and processing for PCR.

### Sample processing

Skin samples in saline were centrifuged at 1500 rpm for 3 min and sediments (20 μl) from these aliquots were individually observed under a light microscope. Microfilariae were identified based on their morphological features that is, in brief, presence of short flattened unsheathed body (mean length 110.1 ± 7.5 μm, width 6.8 ± 1.2 μm) with a rounded head bearing a tiny tooth in the cephalic region [18].

Skin samples were processed for molecular amplification and sequencing of the partial cytochrome oxidase subunit 1 (*cox*1) gene, according to a PCR procedure previously described [4].

### Imaging diagnosis

A MRI study was only conducted in two dogs testing PCR-positive for *O. lupi*, because the other three positive dogs were previously adopted. After a thorough neurological and eye exam, MRI was performed with the two dogs in a sternal recumbent position under general anaesthesia, induced with propofol (Vetofol® 10 mg/ml, 0.5–1 mg/kg as needed) and maintained with isofluorane (Isoflo®, 1.5 % spontaneous ventilation).

Images were acquired by using a 0.2 Tesla MRI machine (ESAOTE VetMR), at slice thicknesses of 4 mm in the dorsal and sagittal planes and 4.5 mm in the transverse plane. T2-weighted images were examined in three planes (dorsal Gradient Echo T2). Transverse FLAIR and T1-weighted images (transverse and dorsal) were also examined with and without contrast (gadoteric acid 0.5 mmol/ml- Dotarem® 0.2 ml/kg). During the recovery time from anaesthesia, the two dogs were also subjected to ocular ultrasonography in which a 10 MHz transducer (ESAOTE MyLab 40) was gently applied to the cornea via a small amount of viscous coupling gel.

### Ethical considerations

The study was carried out in accordance with Spanish Legislation guidelines (Ley 11/2003, Comunidad Autónoma de Andalucía) and the International Guiding Principles for Biomedical Research Involving Animals, issued by the Council for the International Organizations of Medical Sciences.

## Results and discussion

Mean age and weight of the 104 dogs examined were 3.82 years (standard deviation (SD): 2.87) and 25.99 kg (SD: 14.04), respectively. In the population sampled there was a biased proportion towards males (70 %).

Five out of 104 tested dogs (4.8 %) were skin snip-positive for *O. lupi*: two by light microscopy and three by PCR (Table 1), with an overall prevalence of 4.8 % lower than that reported in Portugal (8.3 %) and Greece (8.7 %) [4].

**Table 1:** Detailed results of different techniques used on skin-snip of the five dogs testing positive

Animal ID	Skin samples	
	Microscopy examination	PCR (NTF/NTR) plus sequence analysis
1	NEG	99 % KX132091 *O. lupi* h1
2	NEG	100 % KC686701 *O. lupi* h1
3	*O. lupi*	NEG
4	*O. lupi*	NEG
5	NEG	100 % KC686701 *O. lupi* h1

This is the first report of *O. lupi* in Spain and the first epidemiological study on this helminthic infection, since only one epidemiological study has been undertaken in Greece and Portugal [4] in areas where the presence of *O. lupi* in dogs had been previously recorded. Any of the dogs herein tested presented ocular lesions or other clinical signs, similar to the findings of the previous epidemiological study conducted in Portugal and Greece [4], in which *O. lupi* microfilariae were diagnosed in skin snip sediments of the asymptomatic animals sampled. In the present study, one of the *O. lupi* infected dogs showed neurological abnormalities, although previous studies have not reported this clinical sign in dogs positive to *O. lupi*. However, this condition was described in a 13-year-old boy presenting neurological signs due to aberrant epidural cervical infection, in the spinal canal, by adult nematodes [19]. However, since an aberrant laryngeal location of *O. lupi* was previously reported in a dog from Portugal [19], the dog with neurological signs is referred to as (dog 1, Table 1) and one without those signs as (dog 4, Table 1), were subjected to imaging tests to detect the presence of adults or nodules containing *O. lupi* adults. No granulomatous nodules were detected by this technique although it would have been interesting to examine the remaining animals using imaging techniques.

*O. lupi* microfilariae were detected in skin samples by microscopy examination in two dogs and in three other dogs by PCR diagnosis (*n* = 3). PCR products were sequenced compared to those available in GenBank. Sequences showed a 100 % (*n* = 2) nucleotide identity with those of *O. lupi* in the GenBank database (AN = KC686701). The sequence from another nematode displayed 99 % sequence homology (*n* = 1) with the sequence above, therefore representing a new sequence haplotype (AN = KX132091). It is noteworthy that skin samples from two dogs were microscopy positive for *O. lupi* but PCR negative; this could be because the parasite massively migrated from the skin to saline solution with only the microfilariae being detected in the sediment. Otranto et al. reported a greater number of microfilaria were observed in the sediment when the skin sample was stored for a longer time in the saline solution [15]. On the other hand, three dogs were PCR positive but negative at microscopy for *O. lupi*; this could be explained because microfilariae could not be observed in the sediment due to the presence of low parasite load.

## Conclusion

This first report of *O. lupi* infection in dogs in southern Spain expands the range of geographical distribution of this parasite and sounds an alarm bell for practitioners and physicians working in that area. In an investigation underway, the clinical impacts of infection in affected dogs and the epidemiological factors influencing the presence of this unknown parasite are being examined.

## References

[1] Rodonaja TE (1967). A new species of Nematode, *Onchocerca lupi* n. sp., from *Canis lupus cubanensis*. Soobshchenyia Akad Nauk Gruz SSR.

[2] Széll Z, Sréter T, Erdélyi I, Varga I (2001). Ocular onchocercosis in dogs: aberrant infection in an accidental host or lupi onchocercosis?. Vet Parasitol.

[3] Komnenou AT, Thomas AL, Papadopoulos E, Koutinas AF. Intraocular localization of *Onchocerca lupi* adult worm in a dog with anterior uveitis: A case report. Vet Ophthalmol [Internet]. 2015 Apr; Available from: http://www.ncbi.nlm.nih.gov/pubmed/25929486 10.1111/vop.12277 25929486

[4] Otranto D, Dantas-Torres F, Giannelli A, Latrofa MS, Papadopoulos E, Cardoso L (2013). Zoonotic *Onchocerca lupi* infection in dogs, Greece and Portugal, 2011–2012. Emerg Infect Dis.

[5] Komnenou A, Eberhard ML, Kaldrymidou E, Tsalie E, Dessiris A (2002). Subconjunctival filariasis due to *Onchocerca* sp. in dogs: report of 23 cases in Greece. Vet Ophthalmol.

[6] Komnenou A, Egyed Z, Sréter T, Eberhard ML (2003). Canine onchocercosis in Greece: report of further 20 cases and molecular characterization of the parasite and its Wolbachia endosymbiont. Vet Parasitol.

[7] Hermosilla C, Hetzel U, Bausch M, Grübl J, Bauer C (2005). First autochthonous case of canine ocular onchocercosis in Germany. Vet Rec.

[8] Faísca P, Morales-Hojas R, Alves M, Gomes J, Botelho M, Melo M (2010). A case of canine ocular onchocercosis in Portugal. Vet Ophthalmol.

[9] Tudor P, Turcitu M, Mateescu C, Dantas-Torres F, Tudor N, Bărbuceanu F (2016). Zoonotic ocular onchocercosis caused by *Onchocerca lupi* in dogs in Romania. Parasitol Res.

[10] Labelle AL, Maddox CW, Daniels JB, Lanka S, Eggett TE, Dubielzig RR (2013). Canine ocular onchocercosis in the United States is associated with *Onchocerca lupi*. Vet Parasitol.

[11] Labelle AL, Daniels JB, Dix M, Labelle P (2011). *Onchocerca lupi* causing ocular disease in two cats. Vet Ophthalmol.

[12] Maia C, Annoscia G, Latrofa MS, Pereira A, Giannelli A, Pedroso L (2015). *Onchocerca lupi* Nematode in Cat. Portugal Emerg Infect Dis.

[13] Sréter T, Széll Z, Egyed Z, Varga I (2002). Subconjunctival zoonotic onchocerciasis in man: aberrant infection with *Onchocerca lupi*?. Ann Trop Med Parasitol.

[14] Sréter T, Széll Z (2008). Onchocercosis: a newly recognized disease in dogs. Vet Parasitol.

[15] Otranto D, Dantas-Torres F, Giannelli A, Abramo F, Ignjatović Ćupina A, Petrić D (2013). Cutaneous distribution and circadian rhythm of *Onchocerca lupi* microfilariae in dogs. PLoS Negl Trop Dis.

[16] Franchini D, Giannelli A, Di Paola G, Cortes H, Cardoso L, Lia RP (2014). Image diagnosis of zoonotic onchocercosis by *Onchocerca lupi*. Vet Parasitol.

[17] Grácio AJ, Richter J, Komnenou AT, Grácio MA (2015). Onchocerciasis caused by *Onchocerca lupi*: an emerging zoonotic infection. Systematic review. Parasitol Res.

[18] Mutafchiev Y, Dantas-Torres F, Giannelli A, Abramo F, Papadopoulos E, Cardoso L (2013). Redescription of *Onchocerca lupi* (Spirurida: Onchocercidae) with histopathological observations. Parasit Vectors.

[19] Chen T, Moon K, deMello DE, Feiz-Erfan I, Theodore N, Bhardwaj RD (2015). Case report of an epidural cervical *Onchocerca lupi* infection in a 13-year-old boy. J Neurosurg Pediatr.

